# Comparison of parenchymal and ventricular intracranial pressure readings utilizing a novel multi-parameter intracranial access system

**DOI:** 10.1186/2193-1801-4-10

**Published:** 2015-01-06

**Authors:** Tracey Berlin, Cristina Murray-Krezan, Howard Yonas

**Affiliations:** 16 Department of Neurosurgery, University of New Mexico Hospital, MSC10 5615, 1 University of New Mexico, Albuquerque, NM 87131-0001 USA; 17 Division of Epidemiology, Biostatistics, and Preventive Medicine, Department of Internal Medicine, University of New Mexico School of Medicine, MSC10 5550, 1 University of New Mexico, Albuquerque, NM 87131-0001 USA; 18 Department of Neurosurgery, University of New Mexico School of Medicine, MSC10 5615, 1 University of New Mexico, Albuquerque, NM 87131-0001 USA

**Keywords:** Parenchymal ICP, Ventricular ICP, Intracranial pressure, External ventricular drain, Cerebrospinal fluid, Neurocritical care, Traumatic brain injury, Subarachnoid hemorrhage, InnerSpace Neuro Solutions, Inc, Hummingbird Synergy Ventricular System

## Abstract

**Introduction:**

Both ventricular and parenchymal devices are available for measurement of intracranial pressure (ICP). The Hummingbird^®^ Synergy Ventricular System is a novel device allowing multi-parametric neurological monitoring, including both ventricular and parenchymal ICP. The purpose of this study is to compare the congruence of the device’s ventricular and parenchymal ICP readings.

**Methods:**

This single-center, quantitative, interventional study compared parenchymal and ventricular ICP readings from 35 patients with the Hummingbird^®^ System. If a difference of > ± 3 mmHg existed between an individual patient’s parenchymal and ventricular values, progressive intervention strategies were applied to correct identified issues.

**Results:**

From a total of 2,259 observations, statistical analysis revealed congruence (within ±0-3 mmHg) of 93% of readings comparing parenchymal and ventricular ICP. Of the observations requiring intervention, 58% involved the parenchymal component, 30% involved the ventricular component, and 12% involved both components. Following prescribed interventions, 98% of readings became congruent (within ±0-3 mmHg). The adjusted mean difference between the two methods was -0.95 (95% CI: -0.97,-0.93) mmHg and all mean ICP readings fell between -2 and 2 mmHg.

**Conclusion:**

The Hummingbird^®^ Synergy Ventricular System demonstrates congruence between ventricular and parenchymal ICP measurements within accepted parameters. Interventions required to realign parenchymal and ventricular readings serve as reminders to clinicians to be vigilant with catheter/cable connections and to maintain appropriate positioning of the ventricular drainage system. The results of this study support the recommendation to use the parenchymal ICP component for routine ICP monitoring, allowing dedication of the ventricular catheter to drainage of cerebrospinal fluid (CSF).

## Background

### ICP monitoring and associated waveforms

Prevention and control of increased ICP and maintenance of cerebral perfusion pressure (CPP) are fundamental therapeutic goals for critically ill neuroscience patients (Smith 2008). Both intraventricular and intraparenchymal devices are widely used in clinical practice for measuring ICP. These devices are resource-intensive and carry small but significant risks of infection and hemorrhage (Frattalone and Stevens 2011). ICP monitoring devices measure the sum of the pressures exerted within the cranium by blood, brain, and cerebrospinal fluid (Littlejohns and Bader 2008). They also transmit a waveform, representing the pressure pulse wave being transmitted into the intracranial compartment by systemic hemodynamics. Changes in brain compliance can be seen as alterations in the normal waveform pattern. ICP waveform morphology should be considered when assessing reliability of ICP readings. The type of ICP monitoring device, the location in which it is placed, and the length of time implanted influence device functionality, accuracy, and incidence of complications (Littlejohns and Bader 2008).

### Ventricular ICP monitoring

Historically, the “gold standard” of ICP monitoring has been via surgical insertion of a catheter into the ventricle of the brain (Brean et al. 2006; Schimpf 2012). The ventricular catheter is attached to a fluid-filled external drainage system and, with application of a fluid-filled transducer, is capable of monitoring ICP as well as draining cerebrospinal fluid (CSF) as a therapeutic measure (Zhong et al. 2003; Brain Trauma Foundation 2007). External ventricular drainage systems (EVDs) allow only one mode of operation at a time. By manipulating stopcocks, EVDs can be configured to either monitor ICP or drain CSF, but not perform both simultaneously (Zhong et al. 2003).

For accurate monitoring of ICP via the EVD system, the mounted transducer must be positioned and maintained at the level of the foramen of Monroe or, anatomically, the external auditory meatus (EAM) (Zhong et al. 2003). The accuracy of ICP readings therefore becomes dependent upon patient positioning in relation to the transducer (Zhong et al. 2003; Brain Trauma Foundation 2007). Accuracy of readings also depends upon periodically opening the transducer to atmospheric pressure and returning it to a zero reference point (Schimpf 2012). Hospital protocols differ in the frequency with which EVDs need to be zeroed, but the zeroing procedure typically occurs during each patient care shift, following patient transport, and as a troubleshooting measure when ICP readings or associated waveforms are in question. With each zeroing, the transducer must be exposed to atmosphere, necessitating a break in the sterile integrity of the system through opening of stopcocks and port caps.

Use of the ventricular catheter for ICP monitoring requires continuous nursing observation and intervention to maintain appropriate patient positioning for accurate monitoring (Littlejohns and Bader 2008). With a ventricular catheter, CSF drainage must be periodically interrupted to obtain ICP readings. Patients with limited intracranial compliance may not tolerate interruption of CSF drainage, even for short periods of time (Vender et al. 2011).

Complications related to ventricular catheters include 1%-10% risk of infection and 1%-2% risk of bleeding (Littlejohns and Bader 2008). Ventricular catheters may be difficult to place when there is compression or shift of the ventricles. In cases of a misplaced catheter, the ICP waveform may be dampened and the ICP values inaccurate (Zhong et al. 2003).

### Parenchymal ICP monitoring

Parenchymal ICP monitoring involves placement of a fiberoptic, strain-gauge, or air bladder (pneumatic) device within the brain tissue. While parenchymal monitoring devices do not allow drainage of CSF, their ICP monitoring accuracy is reliable, second only to intraventricular monitoring (Zhong et al. 2003). Parenchymal monitoring devices require calibration and zeroing only once before insertion. Accuracy of readings is not dependent upon patient positioning in relation to the transducer (Zhong et al. 2003; Brain Trauma Foundation 2007). However, without the ability to recalibrate the sensor in situ, parenchymal devices are subject to varying degrees of zero drift over time (Brain Trauma Foundation 2007; Lescot et al. 2011). Complications include infection and bleeding (2%), although the incidence is lower with these devices compared to ventricular catheters (Littlejohns and Bader 2008).

### Comparing ventricular and parenchymal ICP monitoring

There is precedence for comparison of ventricular and parenchymal ICP, yet it is unclear if ventricular and parenchymal pressure differences are expected. Both positive and negative differences are reported by studies comparing ventricular and parenchymal ICP. Review of literature reveals a comparison study of the Spiegelberg parenchymal transducer and ventricular fluid pressure. Utilizing an air bladder technology for parenchymal ICP monitoring, this study showed good agreement between the readings from the Spiegelberg transducer with those from the ventricular drain (Chambers et al. 2001).

Brean et al. (2006) examined differences in pulse amplitude and mean pressure when comparing simultaneous parenchymal and ventricular ICP. Comparison of 218,589 simultaneous single ventricular/parenchymal wave pairs showed marginal differences in pulse pressure amplitude, providing no evidence of a pressure gradient between the ventricular CSF and brain parenchyma (Brean et al. 2006). A somewhat larger difference (expressed as a standard deviation of 6.8 mmHg) in mean ventricular/parenchymal single wave pressures was attributed to hydrostatic pressure differences between the distal end of the EVD and the parenchymal ICP sensor (Brean et al. 2006). In a study published in 2008, Eide compared parenchymal and ventricular pressure readings in patients with documented hydrocephalus and found that the ICP wave amplitude was consistently higher within the ventricular CSF than in the brain parenchyma (Eide 2008).

Lescot et al. (2011) compared ventricular ICP readings to parenchymal readings obtained from both the Codman^®^ and the Pressio^®^ systems. Although there was no statistically significant difference between readings from the parenchymal monitors, the parenchymal ICPs approximated the ventricular CSF pressures by ±7 mmHg. The authors attributed this difference, in part, to measurement of ICP in different levels of the brain depending upon position of the sensor tip and transducer levels. Vender et al. (2011) recently compared hourly intraparenchymal and intraventricular pressure readings in a small cohort of traumatic brain injury patients. They found statistically similar pressure measurements when comparing intraparenchymal to intraventricular monitors.

According to the most recent Brain Trauma Foundation guidelines for management of severe traumatic brain injury (2007), “further improvement in ICP monitoring technology should focus on developing multiparametric ICP devices that can provide simultaneous measurement of ventricular CSF drainage, parenchymal ICP, and other advanced monitoring parameters” (Brain Trauma Foundation 2007).

### The Hummingbird^®^ Synergy Ventricular System

The Hummingbird^®^ Synergy Ventricular System (InnerSpace Neuro Solutions, Inc.) provides uniquely integrated features meeting these criteria and enabling both ventricular drainage and parenchymal ICP monitoring. This cranial access device also allows multi-parameter monitoring by accommodating additional probes to monitor brain tissue oxygen, cerebral perfusion, or cerebral microdialysis. For the purpose of this study, the focus was on the device’s capability for ICP monitoring and ventricular drainage through parenchymal and ventricular components.

In addition to ventricular ICP, a sensor that resides along the ventricular catheter, outside of the ventricle, transmits parenchymal ICP using Air Coupled Transduction (ACT) technology (InnerSpace Neuro Solutions, Inc 2010). ACT technology senses pressure utilizing a proprietary air bladder positioned in the parenchyma. In cases where the ventricles of the brain are inaccessible, this provides a benefit, wherein the catheter can be secured in the parenchyma and used as a parenchymal ICP monitor. This unique technology carries pressure waves from the air column to a reusable transducer housed in the Air Management System (AMS) on the terminal end of the patient monitoring cable. When the AMS is manually cycled, air is removed from the Hummingbird^®^ ICP lumen/sensor membrane and precisely replaced with 32 micro-liters of air, effectively charging the system (InnerSpace Neuro Solutions, Inc 2010). Indicator lights on the AMS housing remind clinicians to re-charge the system every eight hours to maintain the precise amount of air in the system. With this technology, the leveling issues inherent in fluid-coupled systems are eliminated, resulting in precise and positionally-insensitive measurements and an artifact-free, high-fidelity waveform tracing. The AMS patient cable is zeroed from the patient monitor following catheter insertion and initial setup. Re-zeroing is required only as a troubleshooting method, or when the cable becomes disconnected. Neither zeroing nor charging the AMS causes a breach of the sterile integrity of the system. The ability to periodically re-zero the system in situ eliminates the issue of zero drift that is a hallmark of microsensor parenchymal monitors.

Early anecdotal clinical experience of 47 patients utilizing the Hummingbird^®^ Synergy Ventricular System revealed good agreement between ventricular and parenchymal ICP readings and waveform morphologies. Discrepancies in readings typically directed clinicians to issues with the EVD, ranging from the presence of air bubbles or organic matter in the transducer to errors in leveling or a need to re-zero the fluid-coupled system. InnerSpace Neuro Solutions, Inc., manufacturers of the Hummingbird^®^ Synergy Ventricular System, supports earlier findings of Brean et al. (2006), by suggesting that ICP readings from the parenchymal portion of the catheter may differ from ventricular CSF pressure by ±0-3 mmHg due to known hydrostatic pressure differences. In some instances where parenchymal ICP readings were higher than ventricular readings, there was radiographic evidence of focal injury corresponding to potentially higher ICPs in the region surrounding the parenchymal ICP sensor on the catheter.

Chohan et al. (2014) published their institution’s five year experience with use of the Hummingbird^®^ Synergy Ventricular System. In that study, of 275 devices, 251 (91%) were successfully placed on the first attempt, 4 (2%) on the second attempt, and 18 (7%) on >2 attempts. In the literature, the rate of misplaced catheters ranges from 6% to 45% (Chohan et al. 2014). The authors concluded that the system can be placed safely and effectively at the bedside or in the operating room and that it carries a similar or favorable complication profile when compared to other ventricular ICP monitoring systems (Chohan et al. 2014).

With this study, the authors hope to show that the Hummingbird^®^ Synergy Ventricular System transmits parenchymal intracranial pressure (ICP) readings equivalent to ventricular ICP readings within ±3 mmHg (as reported by the manufacturer). Establishing congruence of readings proves reliability of the parenchymal ICP component and allows dedication of the ventricular catheter to cerebrospinal fluid drainage.

## Results and discussion

To our knowledge, this study provides the first clinical data on the accuracy of the Hummingbird^®^ ICP technology compared to standard ventricular ICP monitoring technology. No clinical data have been previously available to support the manufacturer’s claim of congruence of ICP measurements within ±3 mmHg.

Between July 2011 and April 2012, a total of 50 Hummingbird^®^ Synergy Ventricular Systems were inserted in patients in the Neuroscience Intensive Care Unit at the University of New Mexico Hospital, a university-based, Level 1 Trauma Center in Albuquerque, New Mexico. Patients with radiographic confirmation of correct placement of the ventricular catheter were included in the study. Correct placement was defined as verification of the tip of the catheter within the lateral ventricle and the ICP sensing air bladder outside of the ventricle in the brain parenchyma.

Fifteen patients were excluded from the study for various reasons. Seven patients were excluded because the device was not successfully placed in the ventricle of the brain. In these cases, there was no ICP waveform or reading associated with the ventricular catheter, therefore comparison to intraparenchymal ICP was not possible. Three patients were excluded because of difficulty with insertion and suspected rupture of the parenchymal balloon. There was no intraparenchymal waveform or ICP reading, and comparison to ventricular ICP was not possible. Three patients were excluded because of unavailability of the investigator for study enrollment or data collection. Two patients were excluded because of death within four hours of device insertion and inability of investigator to collect data. As a result, 35 patients were successfully enrolled.

Table 1 shows pertinent patient demographics and characteristics. The mean age of patients in the study was 52 years. The majority of subjects (54%) were female and the most common medical diagnosis was subarachnoid hemorrhage (54%). The mean device implant time was 6.9 days with the majority of parenchymal air bladders placed in healthy tissue (74%) on the contralateral side of the injury (86%). Most (83%) ICP recordings were taken with patients’ head of bed elevated to 30 degrees.

**Table 1:** **Patient demographics**/**characteristics**

Sample size, n	35
Age (years), mean ± SD	52 ± 13
Sex, n (%)	
Female	19 (54)
Male	16 (46)
Diagnosis, n (%)	
Subarachnoid hemorrhage	19 (54)
Traumatic brain injury	15 (43)
Intracerebral hemorrhage	1 (3)
Device implant time (days), mean ± SD	6.9 ± 3.0
PAR placement with respect to injured brain, n (%)	
Contralateral	26 (74)
Ipsilateral	9 (26)
PAR placement in tissue, n (%)	
Healthy tissue	30 (86)
Affected tissue	2 (6)
Penumbral region	3 (9)
All ICP values (mmHg), range	
PAR	0-42
EVD	0-40
Mean patient ICP values (mmHg), range	
PAR	5-22
EVD	6-24
Total number of paired ICP readings^1^, n	2258
Number paired device readings per patient, range	8-127
Observations obtained with head of bed at 30°, n (%)	1884 (83)

^1^One EVD value in one subject was not obtained resulting in 2258 EVD and 2259 PAR readings.

### ICP recordings and interventions

Recordings of both ventricular and parenchymal ICP readings obtained from patients with the Hummingbird^®^ Synergy Ventricular System yielded a total of 2,259 observations. Of these, 2,098 (93%) showed congruence between external ventricular drain (EVD) and parenchymal (PAR) ICP readings within ±3 mmHg, therefore requiring no clinical intervention. Differences of 4–8 mmHg between measurements were observed in 167 (7%) readings, and differences ≥9 mmHg were observed in 19 (<1%) readings, leaving a total of 186 observations requiring clinical investigation and possible intervention. Because of patient care activities or changing clinical situations, 161 incongruent events were addressed. Table 2 shows the types of interventions required based on the total number of observations. It also shows the types of interventions involving the PAR component, the EVD, or both, based on the number of events requiring intervention.

**Table 2:** **Intervention types**

Interventions required	No intervention required	PAR source intervention	EVD source intervention	Dual source intervention	Total
	n = 2098	n = 93	n = 49	n = 19	2259
	93%	4%	2%	1%	100%
Intervention types based on total required		PAR	EVD	Dual	Total
		n = 93	n = 49	n = 19	161
		58%	30%	12%	100%

Interventions required based on the total number of observations. Of the total number of observations, 93% required no intervention, meaning that PAR and EVD readings were congruent.

Intervention types based on total events requiring intervention. Of the total number of observations, 161 (7%) required intervention. Of these, the majority (58%) of interventions required to attain congruence of PAR and EVD reading involved the PAR source.

Following device assessment, a total of 177 primary interventions were performed; 109 involved the PAR and 68 involved the EVD. As seen in Table 3, the most frequent primary intervention for the PAR was recharging the AMS, followed by zeroing the AMS and tightening of connections. Using the EVD, the most frequent primary intervention involved leveling the transducer, zeroing it, and then flushing it. After primary intervention, 106 (60%) comparison readings demonstrated congruence of ICP values within ±3 mmHg. A total of 71 secondary interventions were required and performed. The most frequently used secondary PAR intervention was charging the AMS (91%), whereas the most frequently used secondary EVD intervention was zeroing the transducer (88%). Using prescribed interventions, 98% of EVD and PAR readings became congruent (within ±0-3 mmHg) following a secondary intervention. PAR and EVD reading agreement following primary and secondary intervention is displayed in Table 4.

**Table 3:** **Most frequent primary interventions associated with each ICP source**

Primary interventions for PAR	Recharging AMS	Zeroing AMS	Tightening connections
	n = 56	n = 48	n = 5
	51%	44%	5%
Primary interventions for EVD	Leveling transducer	Zeroing transducer	Flushing transducer
	n = 61	n = 5	n = 2
	90%	7%	3%

**Table 4:** **Agreement between parenchymal and ventricular readings following primary and secondary interventions**

	Primary intervention	Secondary intervention
Number of Interventions Performed	177	71
Number of Readings Becoming Congruent After Intervention Type	106	69
Percentage of Readings Becoming Congruent After Intervention Type	60%	98%

The mean PAR ICP reading among the 35 subjects was 10.8 (SD = 3.9) mmHg, with a range of 5–22 mmHg. The mean EVD ICP reading was 11.7 (SD = 3.8) mmHg with a range of 6–24 mmHg. The overall mean difference of the 2248 paired measurements was -1.0 (SD = 1.8) mmHg, with PAR consistently lower than EVD. Mixed linear modeling of the paired differences revealed a significant effect for the repeated measures (all p < 0.05), as well as for intervention, age, implant time, type of injury, the order of the observations; and three interactions: implant time × type of injury, implant time × order of observations, and type of injury × order of observations. Related statistics have been omitted as the aim is to show agreement between measurements, not find predictive factors of the differences. After adjusting for these covariates, the mean difference between the two methods was well within 3 mmHg of each other [adjusted mean difference = -0.95 (95% CI: -0.97,-0.93) mmHg]. While the mean difference between device measurements was not negligible, the difference was less than 1 mmHg. After fitting independent mixed linear models for each of PAR and EVD with the same covariates previously described, the adjusted mean ICP for PAR was 10.8 mmHg (95% CI: 10.6,11.1 mmHg) and for EVD was 11.8 mmHg (11.6,12.0 mmHg).

**Figure 1:**
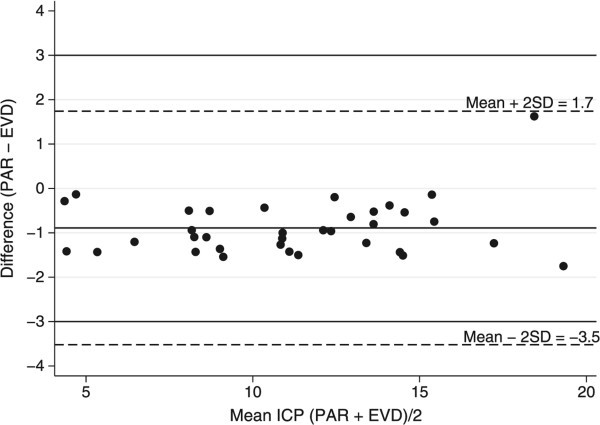
**Bland**-**Altman plot of the difference between mean PAR and mean EVD ICP measurements against the mean of PAR and EVD in each patient in the study.** This shows that, on average, measurements from each method fell within ±3 mmHg of each other.

To further support our findings, we used the Bland-Altman method (Bland and Altman 1999; Myles and Cui 2007) as described in Methods – Statistical Analysis to graphically present the agreement between the two ICP measurement methods. Figure 1 presents the mean difference (bias) between PAR and EVD versus the mean of the two methods along with the 95% limits of agreement adjusted for the repeated measurements and covariates mentioned above. This approach indicates agreement when the difference between the measurements falls between -3.5 and 1.7 mmHg. The data reported here had no outliers beyond these 95% limits of agreement, and all observations fell within ±3 mmHg as specified by the manufacturer.

### Limitations

Several limitations apply to this study. First, ICP comparisons were performed during an 8–10 hour period each day when the Registered Nurse (RN) investigator was present in the clinical environment. Therefore, the data do not represent all measures within a 24-hour period. Next, a second investigator was present to validate data collection results and interventions on the first five subjects. This was done to ensure inter- and intra-rater reliability and minimize bias. However, other than verbal inter-rater agreement, no statistical reliability testing was performed, and data were not collected for this purpose. Finally, the need for intervention to improve congruence of parenchymal and ventricular ICP may be linked more to the skill level and experience of the bedside nurse in managing device technology than to device capability. The level of nursing experience or skill mastery was not considered in the study design.

## Conclusion

The Hummingbird^®^ Synergy Ventricular System demonstrates congruence between parenchymal and ventricular ICP measurements within accepted parameters. Interventions required to realign PAR and EVD readings serve as reminders to clinicians to be vigilant with catheter/cable connections and to maintain appropriate positioning of the ventricular drainage system. The results of this study support the accuracy of parenchymal ICP readings and their congruence with ventricular ICP readings obtained from the Hummingbird^®^ Synergy Ventricular System. The results of this study also support the recommendation to use the PAR ICP sensor for routine ICP monitoring, allowing dedication of the ventricular catheter to drainage of cerebrospinal fluid.

## Methods

### Protection of subjects

There were no risks associated with this study. Patients received standard of care for the intracranial monitoring device and standard ICP management. Following expedited review, study approval was obtained from the University of New Mexico Human Research Review Committee (HRRC). The requirements for informed consent and HIPAA Authorization Addendum were waived.

### Study design

For all subjects enrolled in the study, verification of Hummingbird^®^ Synergy Ventricular System placement was confirmed by radiographic imaging via CT Scan. This is standard of care for patients receiving this device. Subjects did not undergo any additional radiographic exams or diagnostic testing because of participation in the study.

Data for direct comparison of ventricular and parenchymal ICP were gathered using a data collection tool designed for this study. Parenchymal (PAR) and ventricular (EVD) ICPs were recorded every one to two hours (as indicated by clinical condition) for an interval of 8 to 10 hours each day of the implant period during which the RN investigator was clinically present. Values were recorded with the patient at rest in a position with the head of bed elevated at 30 degrees. If the patient’s clinical condition contraindicated positioning at 30 degrees, then the positioning determined to be therapeutic for that patient was used and documented. Parenchymal and ventricular waveforms were assessed for adequacy and congruency. If the ventricular drainage system had been open to allow drainage of CSF, then the drainage portion was closed, the transducer accessed, and ICP recorded after a two-minute equilibration period.

The manufacturer reports an expected variance of parenchymal and ventricular ICP by ±0-3 mmHg. If parenchymal and ventricular ICPs varied by more than ±3 mmHg, the Hummingbird^®^ System and EVD were examined for issues that might contribute to inaccurate values or poor waveform morphology. These issues include air bubbles or organic matter in the EVD transducer, improper leveling of the EVD with the EAM, cable malfunction, scheduled re-charging of Hummingbird^®^ AMS, or other reasons.

### Possible interventions

Interventions within the scope of nursing practice were performed by the RN investigator to correct identified issues. The intervention strategy was based upon device assessment and waveform morphology. Interventions for the parenchymal sensor included the following: the AMS was charged, the ACT ICP was zeroed, the connections tightened, or the cable changed. Interventions for the ventricular catheter included the following: the EVD transducer was leveled or zeroed, the transducer or tubing was flushed to clear out air bubbles, blood, or debris, or the transducer or cable was changed. If ICPs remained incongruent following a single intervention, additional interventions were attempted. After intervention(s) were performed, PAR and EVD ICPs were re-recorded and the required intervention(s) documented. Waveform morphology was again assessed and documented.

### Statistical analysis

A power analysis was performed to determine the adequate sample size necessary for determining whether PAR and EVD ICP measurements were equivalent to within ±3 mmHg of each other, as reported by the manufacturer. Based on preliminary data, the mean (SD) difference over the course of therapy was expected to be approximately 2.0 (2.0) mmHg. Based on those assumptions, a sample size of n = 35 was required to obtain 95% power to determine that the mean difference would fall within the ±3 mmHg margin previously reported. The power analysis was performed in PASS 11 (Hintze 2011).

Descriptive statistics were used to summarize the data. The differences for PAR and EVD ICP measurements were calculated at each time point for every subject and the differences were assessed for normality. A linear mixed model with unstructured covariance was fitted in SAS PROC MIXED to the differences of paired PAR and EVD ICP readings to account for the within-subject variance of the repeated measures and adjust for the following potential covariates: whether an intervention was performed, type of injury, implant side, age, gender, implant length of time, order of the observations, and all two-way interactions. The adjusted mean difference and 95% confidence interval were calculated from this model. A repeated measures multivariate analysis of variance model was fitted to both EVD and PAR ICP readings simultaneously and the same covariates as in the first model were found to be statistically significant. The adjusted mean estimates and their 95% confidence intervals for PAR and EVD measurements were calculated from this model.

The Bland-Altman method (Bland and Altman 1999) for measuring agreement in unequal numbers of replicates was used to calculate the 95% limits of agreement between the two ICP measurement methods. In general, the limits of agreement are calculated as
1


where  is the mean difference of the methods and  is the sample standard deviation of . When data fall within these limits, the methods are generally considered to be in agreement. Following their procedure, Bland and Altman (1999) define the calculation of the square of  (sample variance of the mean difference) for repeated measures with unequal replicates as
2


where  is the number of subjects in the study,  and  are the number of repeated measures for each subject with each method, and  and  are the within-subject variance estimates of PAR and EVD, respectively. However, to better capture the variability due to the repeated measures and unequal replicates, the calculations of  and  were modified by adjusting for the between-subject variances within EVD and PAR measurements, using a method described by Myles and Cui (2007). This was accomplished by fitting two linear mixed models with unstructured covariance to each of PAR and EVD ICP with the independent variables found to be significant from the model of the paired ICP differences described above in addition to a variable of the mean PAR-EVD ICP value at each reading. From these models, the covariance parameter estimates  and  were obtained and applied to [2] for the calculation of the estimated variance of the difference for the 95% limits of agreement. The square root of  was calculated and then substituted into [1] and the limits were obtained. Statistical significance was held at α = 0.05. All analyses were performed in SAS version 9.4 (SAS Institute 2012) and Stata 13/SE (Stata Corp 2014).

## Abbreviations

ACT: Air Coupled Transduction
AMS: Air Management System
CPP: Cerebral perfusion pressure
CSF: Cerebrospinal fluid
EAM: External auditory meatus
EVD: External ventricular drain
HRRC: Human Research Review Committee
ICP: Intracranial pressure
PAR: Parenchymal.
